# The Effect of Latent *Toxoplasma gondii* Infection on the Immune Response in HIV-Infected Patients

**DOI:** 10.1155/2015/271842

**Published:** 2015-07-12

**Authors:** Ondrej Beran, Petr Kodym, Marek Maly, Alzbeta Davidova, Gabriela Reinvartova, David Jilich, Michal Holub, Hanus Rozsypal

**Affiliations:** ^1^Department of Infectious and Tropical Diseases, First Faculty of Medicine, Charles University in Prague and Na Bulovce Hospital, Budínova 2, 180 81 Prague, Czech Republic; ^2^Department of Infectious Diseases, First Faculty of Medicine, Charles University in Prague and Military University Hospital Prague, Prague, Czech Republic; ^3^National Reference Laboratory for Toxoplasmosis, National Institute of Public Health in Prague, Czech Republic; ^4^Department of Biostatistics, National Institute of Public Health in Prague, Czech Republic

## Abstract

A relationship between latent toxoplasmosis and the immune system during HIV disease is poorly understood. Therefore, the aim of this follow-up study was to characterize immunological parameters in HIV-infected patients with latent toxoplasmosis and noninfected individuals. A total of 101 HIV-infected patients were enrolled in the study. The patients were classified into two groups based on anti-*Toxoplasma gondii* antibodies: a group of 55 toxoplasma-positive persons (TP) and a group of 46 toxoplasma-negative persons (TN). Absolute counts of several lymphocyte subsets decreased in the TP group, namely, T cells (*p* = 0.007), B cells (*p* = 0.002), NK cells (*p* = 0.009), CD4 T cells (*p* = 0.028), and CD8 T cells (*p* = 0.004). On the other hand, the percentage of CD8 T cells expressing CD38 and HLA-DR significantly increased during the follow-up in the TP group (*p* = 0.003, *p* = 0.042, resp.) as well as the intensity of CD38 and HLA-DR expression (MFI) on CD8 T cells (*p* = 0.001, *p* = 0.057, resp.). In the TN group, analysis of the kinetics of immunological parameters revealed no significant changes over time. In conclusion, the results suggest that latent *T. gondii* infection modulates the immune response during HIV infection.

## 1. Introduction

*Toxoplasma gondii* is an intracellular protozoon causing one of the most prevalent infections. Apart from humans,* T. gondii* infects various mammals and birds, with small felids playing a major role as definitive hosts of the parasite. Humans are infected either by consumption of undercooked meat containing a tissue cyst or by ingestion of oocysts shed by the definitive host in feces. In women infected during pregnancy* T. gondii* infection can lead to vertical transmission and fetal infection [1, 2].

The primary infection in immunocompetent individuals is mostly asymptomatic or can be manifested as lymphadenopathy and is usually followed by a lifelong latent infection. However, from this state of latency* T*.* gondii* infection may be reactivated as a result of immune disorders [3]. Despite the availability of effective antiretroviral therapy, toxoplasmosis is the most important opportunistic infection in patients infected with human immunodeficiency virus (HIV) and can manifest as a potentially life threatening toxoplasmic encephalitis [4, 5].

Following infection with* T. gondii* different immune cells were shown to have a role in host resistance to this organism [6]. T cells and natural killer (NK) cells help to control the initial infection by production of interferon- (IFN-) *γ* and interleukin- (IL-)12. Long-term host resistance is provided by T and B cells. However, the mechanisms of immune surveillance are not fully understood. Regarding the influence of latent toxoplasmosis on humans, several effects have been documented. Latent toxoplasmosis increases the risks of Parkinson's disease [7], behavioral changes [8–10], or autoimmune diseases [11, 12]. Moreover, latent* T. gondii* infection may lead to immune suppression in both mice and humans [13, 14]. Despite many studies focusing on the relationship of toxoplasmosis reactivation and HIV infection, there is a lack of knowledge about the association between latent toxoplasmosis and activation of the immune system during HIV disease.

Therefore, the aim of the study was to characterize and compare different immunological parameters, including expression of activation markers on CD8 T cells of HIV-infected patients with latent toxoplasmosis and HIV-infected individuals without latent toxoplasmosis during a one-year follow-up.

## 2. Patients and Methods

### 2.1. Study Population

A total of 101 HIV-positive patients (sex ratio M/F, 94/7; mean age, 43; range, 26–74 years) registered at the AIDS Centre at Na Bulovce Hospital in Prague were enrolled in the study between December 2012 and April 2013. This prospective study was conducted in accordance with the Helsinki Declaration as revised in 2000 after obtaining the approval from the local ethics committee. A written informed consent was obtained from all study participants. The patients were randomly selected in order to represent the entire cohort of HIV patients treated at the centre. Based on anti-*T. gondii* antibodies, the patients were classified into two groups: a group of toxoplasma-positive (TP), which comprised 55 seropositive patients, and a group of 46 toxoplasma-negatives (TN). In Table 1, demographic and clinical parameters at the time of enrollment are presented for both groups. According to the CDC classification, 14 (14%) patients were classified with the AIDS stage of the HIV disease [15]. The percentages of patients with AIDS stage did not differ between the groups and these patients were not excluded from the analysis. The patients were evaluated regarding presence of important coinfections, that is, hepatitis C, hepatitis B, and cytomegalovirus infection (positive serology anti-HCV, anti-HBcAg IgG, and anti-CMV). The statistical analysis demonstrated that the TP and TN groups were not different as far as the presence of these coinfections is concerned. Patients with clinical and laboratory signs of active coinfections were excluded from the study. Regarding combined antiretroviral therapy (cART), 49 patients (89%) were treated in the TP group and 43 patients (93%) in the TN group. The percentages of patients with different treatment protocols are also presented in Table 1. Plasma viral load values were undetectable or very low in a majority of patients. Blood samples for immunological analyses were collected from all patients upon enrollment and after a one-year interval.

### 2.2. Flow Cytometric Analysis

Routine immunophenotyping (T cells, B cells, CD4 T cells, CD8 T cells, and NK cells) and an analysis of the surface expression of activation markers (CD38 and HLA-DR) on CD8 T cells were done from EDTA-treated peripheral blood using monoclonal antibodies (Ab) (Becton Dickinson, BD, Germany) [16]. Analysis was performed by six-color flow cytometry (FACSCanto, BD, USA) using FACSDiva software (BD). Expression is presented as mean fluorescence intensity (MFI).

### 2.3. Toxoplasma Serology Testing

During the follow-up, the complement-fixation test (CFT) and IgM/IgG ELISA test were used for detection of anti-*T*.* gondii* antibodies [17, 18]. Positive results for CFT were defined as titers of ≥1 : 4 and IgG ELISA absorbance ≥0.25; IgM was positive at a positivity index (absorbance of tested sample/absorbance of cut-off control) of >1.1.

### 2.4. Statistical Analysis

Geometric means together with corresponding 95% confidence intervals (95% CI) were calculated to characterize the location and variability of the analyzed variables in the groups. The analysis of mean change during follow-up in individual groups was based on Student's paired *t*-test. The comparison of the change magnitude between TP and TN group was performed using an analysis of variance with the interaction term between the group and time. The tests were applied to logarithmically transformed data. All statistical tests were evaluated as two-sided at a significance level of 0.05. Stata release 9.2 (Stata Corp. LP, College Station, TX) statistical software was used for statistical analysis.

## 3. Results

**Figure 1:**
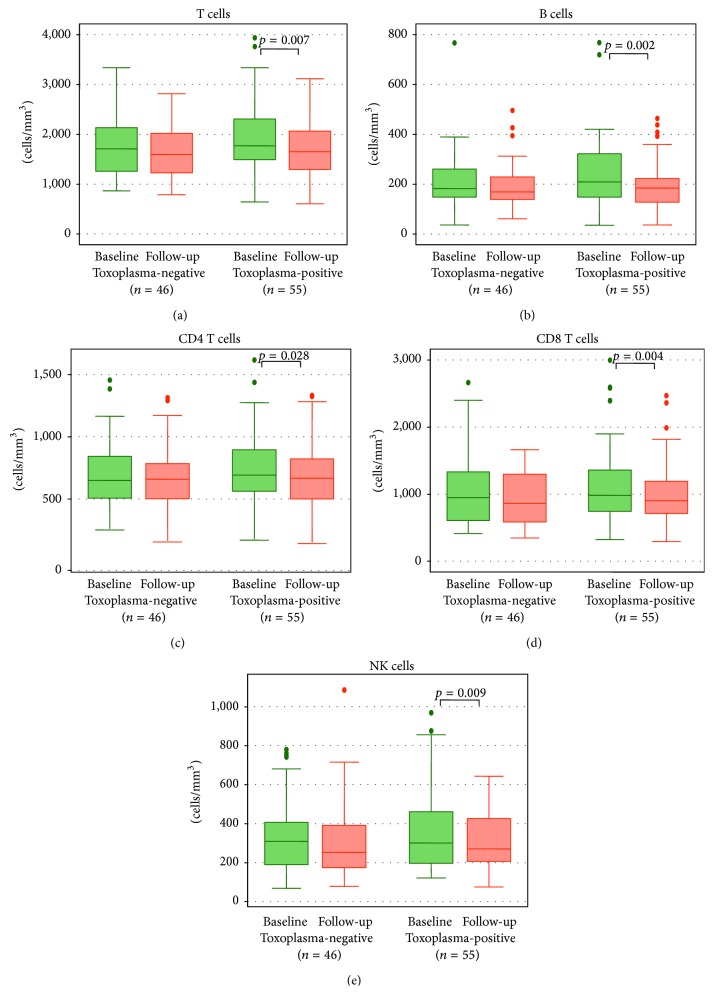
The kinetics of absolute counts of T cells (a), B cells (b), CD4 T cells (c), CD8 T cells (d), and NK cells (e) in toxoplasma-negative and toxoplasma-positive patients during the study. The results document the decrease of all lymphocytes subsets in the toxoplasma-positive group. Data in box plots are expressed as medians and interquartile range.

**Figure 2:**
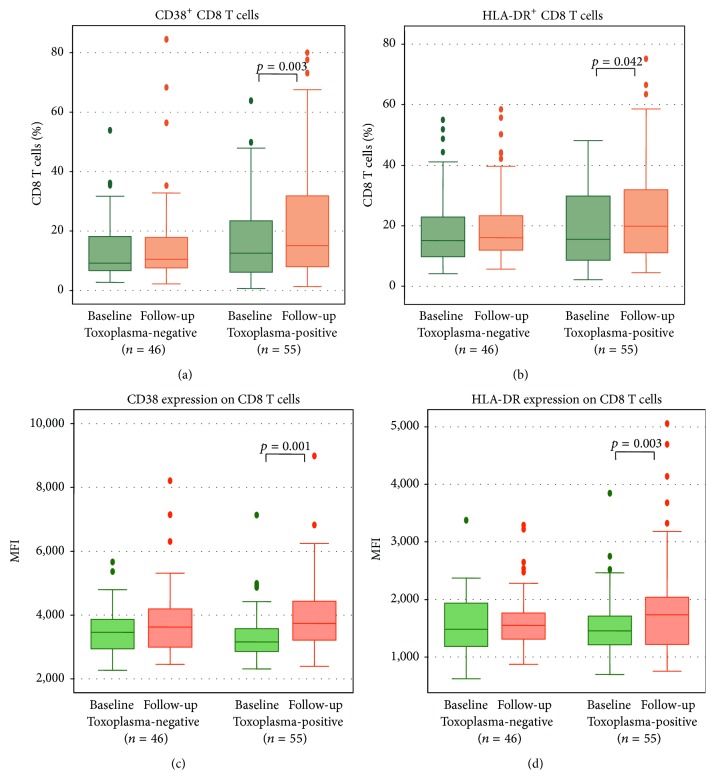
The kinetics of percentage of CD38^+^ and HLA-DR^+^ CD8 T cells (a, b) and mean fluorescence intensity (MFI) of CD38 and HLA-DR on CD8 T cells (c, d) in toxoplasma-negative and toxoplasma-positive patients over the study. CD8 T cells are gated as CD3^+^/CD8^+^ cells. *p* values denote significant differences in the toxoplasma-positive group. Data in box plots are expressed as medians and interquartile range.

During the study follow-up, none of the 46 initially TN patients were infected with* T. gondii* and seroconverted. The comparison of laboratory parameters between the TN and TP groups at the time of enrollment and after a one-year follow-up is presented in Table 2. Interestingly, significant changes of evaluated parameters were observed only in the group TP (Figures 1(a)–1(e)). The decrease was found in absolute counts of T cells (*p* = 0.007), B cells (*p* = 0.002), NK cells (*p* = 0.009), CD4 T cells (*p* = 0.028), and CD8 T cells (*p* = 0.004). On the other hand, as shown also in Figures 2(a)–2(d), the percentage of CD8 T cells expressing CD38 and HLA-DR antigens significantly increased in the TP group (*p* = 0.003, *p* = 0.042, resp.) similar to the intensity of CD38 and HLA-DR expression (MFI) on CD8 T cells (*p* = 0.001, *p* = 0.057, resp.). In the TN group, the analysis of the kinetics of immunological parameters revealed no significant changes over time.

## 4. Discussion

In this prospective follow-up study, we characterized the immune response in HIV-infected patients latently infected with* T. gondii *in comparison with TN individuals during a one-year follow-up. Latent toxoplasmosis status was defined as the presence of anti-*T. gondii* antibodies without any clinical symptoms. During the study, in none of the TP patients latent toxoplasmosis had been reactivated. This finding reflects the fact that the majority of patients had good immunological parameters and only a few individuals equally distributed in both groups were enrolled with HIV infection in stage 3 (AIDS). Moreover, the incidence of reactivation is quite low and was documented to be 3.4 cases per 1000 anti-*T*.* gondii* positive HIV-infected patients per year in the Czech Republic [1]. The prevalence of latent toxoplasmosis in HIV-infected persons in the Czech Republic is 40.2%. The decline of the cases with reactivation observed after 1996 was due to improved efficacy of antiretroviral therapy restoring cell mediated immunity including recovery of anti-*T*.* gondii* specific CD4 and CD8 T cell responses [19]. However, previous studies documented that restoration of the immune system including antigen-specific responses in patients treated with cART is not complete [20, 21]. Despite the studies describing the effect of HIV-induced immunodeficiency on toxoplasmosis reactivation, the impact of latent* T. gondii* infection on HIV disease progression and immune parameters is not clear.

We observed the decrease in the majority of lymphocyte subset counts in the group of HIV-infected patients with latent* T*.* gondii* infection. This finding is in line with a previous study in HIV-negative persons, where male patients with latent toxoplasmosis had lower B cells, NK cells, and monocytes [14]. CD4 T cells are critical in the immune defense against toxoplasmosis in HIV infection and the reactivation of* T*.* gondii* infection is associated with the decline of this T cell subset [22]. In our study the mean CD4 counts were above 500 cells/mm^3^ in both groups during the follow-up period, and the mean decrease in the TP group was 56 cells/mm^3^ compared to 36 cells/mm^3^ in the TN group. The observed decline of CD4 cell count can be attributed to HIV progression but partly also to the potential effect of latent toxoplasmosis. In a previous study of HIV-negative individuals CD4 cell count was not influenced by latent toxoplasmosis, but the discrepancy may also reflect the fact that the enrolled HIV-negative patients had different immunopathological conditions [14].

Although CD4 T cells play a key role in the immunity against* T. gondii *as important producer of IFN-*γ* [23], CD8 T cell and NK cell deficiencies were previously demonstrated to contribute to toxoplasmosis reactivation [1, 24]. In a mouse model, CD8 T cells showed protective effect in the initial stages of reactivation of* T*.* gondii* infection [25]. Specific CD8 T cells play a synergistic role with CD4 T cells in IFN-*γ* production and protective immunity against* T*.* gondii* infection [26]. Similar to other lymphocyte subsets, reduced numbers of NK cells were previously demonstrated in HIV-infected persons with toxoplasmosis reactivation [1]. The trend of NK cell and CD8 cell counts decline observed in the TP group was not associated with toxoplasmosis reactivation in the patients. However, the association with the presence of* T*.* gondii* latent infection may have had an influence on a more rapid long-term progression of HIV disease.

In our study, the percentages of activated CD8 T cells characterized by the expression of CD38 and HLA-DR increased in the TP group during the follow-up. Similar to the lymphocyte subset counts, these changes were not observed in TN patients. Previous studies demonstrated that increased expression of CD38 and HLA-DR markers on T cells correlates with disease progression and depletion of CD4 T cells, better than the level of the viral load [27]. Also, many studies demonstrated that MFI of CD38 on CD8 T cells represents a reliable laboratory marker for routine monitoring of HIV-infected patients [28]. Our results may indicate a possible modulation of nonspecific CD8 T cell activation by latent toxoplasmosis. This effect may be indirect and the trend of the CD8 T cell activation increase may be a result of the mild decrease of CD4 cell count observed during the follow-up. In previous studies, the immunomodulatory effect of latent toxoplasmosis in HIV-negative humans was documented and it decreased with the duration of the infection [14].

## 5. Conclusions

This study presents new findings about the role of latent* T*.* gondii* infection in modulation of the immune responses during HIV infection. The results suggest that latent toxoplasmosis has an effect on the kinetics of lymphocyte subset counts and surface expression of immune activation markers in HIV-infected persons. The observed changes are mild and further studies are warranted to elucidate the impact of highly prevalent* T. gondii* infection on the complex immunopathogenesis of HIV infection.

## Figures and Tables

**Table 1:** Demographic and clinical characteristics from 46 toxoplasma-negative and 55 toxoplasma-positive HIV-infected patients upon the time of enrollment.

Parameter	Toxoplasma-negative	Toxoplasma-positive	*p* value
Age (arithmetic mean ± SD)	42 ± 9	44 ± 11	0.382
Sex (male/female)	43/3	51/4	1.000
Previous AIDS diagnosis (%)	13	15	1.000
Time since first positive HIV test (years)^*∗*^	9 (8–10)	9 (8–11)	0.856
Time since cART initiation (years)^*∗*^	5.7 (4.5–7.3)	5.4 (4.3–6.9)	0.766
Coinfections			
HBV (%)	41	35	0.539
HCV (%)	13	13	1.000
CMV (%)	87	89	0.767
Antiretroviral treatment (cART)			0.759
2 NRTI + 1 PI (%)	43	45	
2 NRTI + 1 NNRTI (%)	35	27	
2 NRTI + 1 II (%)	9	13	
3 NRTI (%)	9	6	
None (%)	4	9	

SD, standard deviation; AIDS, acquired immunodeficiency syndrome; HIV, human immunodeficiency virus; cART, combined antiretroviral therapy; HBV, hepatitis B virus; HCV, hepatitis C virus; CMV, cytomegalovirus; NRTI, nucleoside reverse transcriptase inhibitors; PI, protease inhibitors; NNRTI, nonnucleoside reverse transcriptase inhibitors; II, integrase inhibitors.

^*∗*^Values are presented as geometric means and 95% confidence intervals.

**Table 2:** Comparison of laboratory data from toxoplasma-negative and toxoplasma-positive HIV-infected patients upon the time of enrollment and after a one-year follow-up.

Parameter	Toxoplasma-negative (baseline)	Toxoplasma-negative (follow-up)	*p*	Toxoplasma-positive (baseline)	Toxoplasma-positive (follow-up)	*p*
T cells (cells/mm^3^)	1669 (1510–1845)	1575 (1435–1729)	0.109	1814 (1656–1988)	1629 (1477–1796)	0.007
CD4 T cells (cells/mm^3^)	666 (602–737)	630 (559–710)	0.212	688 (614–771)	632 (561–713)	0.028
CD8 T cells (cells/mm^3^)	904 (781–1046)	839 (730–964)	0.052	1002 (880–1141)	882 (769–1012)	0.004
B cells (cells/mm^3^)	184 (156–216)	175 (154–200)	0.361	205 (177–239)	174 (151–201)	0.002
NK cells (cells/mm^3^)	290 (244–344)	261 (220–309)	0.063	317 (273–367)	280 (243–323)	0.009
CD38^+^ CD8 T cells (%)	11 (9–13)	12 (10–15)	0.144	12 (9–15)	16 (13–20)	0.003
CD38 expression on CD8 T cells (MFI)	777 (667–905)	941 (777–1138)	0.036	844 (712–999)	1184 (964–1453)	0.001
HLA-DR^+^ CD8 T cells (%)	15 (13–19)	17 (14–20)	0.125	16 (14–20)	19 (16–23)	0.042
HLA-DR expression on CD8 T cells (MFI)	258 (209–317)	281 (224–353)	0.174	266 (213–333)	333 (252–438)	0.003

MFI, mean fluorescence intensity.

Values are presented as geometric means and 95% confidence intervals.

## Abbreviations

AIDS: Acquired immunodeficiency syndrome
cART: Combined antiretroviral therapy
CFT: Complement fixation test
CMV: Cytomegalovirus
HBV: Hepatitis B virus
HCV: Hepatitis C virus
HIV: Human immunodeficiency virus
IFN: Interferon
IL: Interleukin
NK: Natural killer
MFI: Mean fluorescence intensity
TN: Toxoplasma-negative
TP: Toxoplasma-positive.
